# P-1295. Meningococcal Disease in Children in Argentina: A 4-year Active Sentinel Hospital Surveillance Study during the Post-Meningococcal Vaccination Era: A Difficult Impact Analysis

**DOI:** 10.1093/ofid/ofae631.1476

**Published:** 2025-01-29

**Authors:** Angela Gentile, Julia Bakir, Mara N Maydana, Juan C Morales, Gabriela Ensinck, Adriana Ernst, Pablo A Melonari, Ana C Cardozo, Alejandro Santillan Iturres, Mauricio Santos, Adriana Efron

**Affiliations:** Ricardo Gutierrez Children's Hospital, Buenos Aires, Ciudad Autonoma de Buenos Aires, Argentina; Ricardo Gutierrez Children's Hospital, Buenos Aires, Ciudad Autonoma de Buenos Aires, Argentina; Hospital de Niños Sor Ludovica, La Plata, Buenos Aires, Argentina; Hospital de Niños Sor Ludovica, La Plata, Buenos Aires, Argentina; Hospital de Niños Victor Vilela, Rosario, Santa Fe, Argentina; Hospital de Niños Victor Vilela, Rosario, Santa Fe, Argentina; Hospital Pediatrico Humberto Notti, Mendoza, Mendoza, Argentina; Hospital Pediatrico Juan Pablo II, Corrientes, Corrientes, Argentina; Hospital de Niños Eva Peron, Catamarca, Catamarca, Argentina; INEI-ANLIS “Dr. Carlos G. Malbrán”, CABA, Ciudad Autonoma de Buenos Aires, Argentina; INEI-ANLIS “Dr. Carlos G. Malbrán”, CABA, Ciudad Autonoma de Buenos Aires, Argentina

## Abstract

**Background:**

In 2017, MenACWY-CRM197 vaccine was introduced in the national immunization program (NIP) in Argentina (2+1 schedule, +1 dose at 11 years). In the pre-vaccination period (preVp, 2012-2015), the meningococcal disease (MD) burden had been assessed.

The objectives of this study were: to evaluate the impact of the intervention on the burden of MD in ≤15 years during a post-vaccination period (postVp, January 2020 to December 2023) and to describe the clinical-epidemiological-bacteriological pattern of all confirmed MD cases of postVp versus preVp.

**Methods:**

Active hospital surveillance in 6 pediatric sentinel units in Argentina. MD discharge rates per 10,000 hospital discharges between preVp and postVp was compared.

**Results:**

In postVp, of 186,257 hospitalized patients, 1047 (0.6 %) had suspected meningitis or MD, of which 9.1 % (95/1047) had acute bacterial meningitis (ABM) and 1.1 % (11/1047) meningococcemia; 79 % (75/95) of ABM cases were confirmed by culture, corresponding 8.0 % (6/75) to *Neisseria meningitidis* (Nm). In addition, in 2 out of 11 meningococcemia cases a Nm was confirmed by culture. 41.4 % (12/29) of MD culture-negative cases were confirmed by PCR. Thus, there were a total of 20 MD cases, a discharge rate of 1.1 (0.7-1.7) and a reduction of 78.9 % compared to the preVp. Of the 20 MD cases in the postVp, median age was 38 months (IQR 26-95), 13 had MenACWY-CRM197. Microbiological/molecular diagnosis was made in blood/serum (9; 40.9 %) and CSF (13; 59.1 %). Capsular group was identified in 14 samples (B:13; C:1).

**Conclusion:**

After the MenACWY-CRM197 vaccine introduction in the NIP, the burden of MD in ≤15 years was reduced almost 80%. The predominant serogroup was B.

These findings could be interpreted as an effect of the vaccine, but it is necessary to also take into account the biases inherent in vaccine impact studies (COVID-19 pandemic, lockdown measures, natural fluctuations in the incidence of MD, among others).

**Disclosures:**

**All Authors**: No reported disclosures

